# Effect of Temperature and Photoperiod on Development, Survival, and Growth Rate of Mealworms, *Tenebrio molitor*

**DOI:** 10.3390/insects13040321

**Published:** 2022-03-24

**Authors:** Stephan Eberle, Lisa-Marie Schaden, Johannes Tintner, Christian Stauffer, Martin Schebeck

**Affiliations:** 1 Department of Forest and Soil Sciences, Institute of Forest Entomology, Forest Pathology and Forest Protection, University of Natural Resources and Life Sciences Vienna, A-1190 Vienna, Austria; eberle.stephan@web.de (S.E.); martin.schebeck@boku.ac.at (M.S.); 2 LarveMe (Formerly DieWurmfarm), A-9433 St. Andrä, Austria; office@larveme.com; 3 Department of Material Sciences and Process Engineering, Institute of Physics and Materials Science, University of Natural Resources and Life Sciences Vienna, A-1190 Vienna, Austria; johannes.tintner@boku.ac.at

**Keywords:** temperature, photoperiod, development, entomophagy, *Tenebrio molitor*, mealworm

## Abstract

**Simple Summary:**

The mealworm, i.e., larvae of *Tenebrio molitor*, has recently been used as a substitute for conventional meat, as it has several advantages, e.g., a lower environmental impact. Knowledge of the effects of temperature and photoperiod on mealworm development is crucial to increase farming efficiency, and to contribute to a sustainable production process. As such data is scarce, we tested the effects of three different temperatures, in combination with three photoperiods, on mealworm survival, development, and growth. We found that temperature strongly affects the survival rate, developmental time, and growth rate of *T. molitor* larvae. Furthermore, photoperiod influences the developmental time and growth rate. The highest survival rates and growth rates, and shortest developmental times, were observed at 25 and 30 °C at constant darkness. These results are important to improve the mass rearing of mealworms for a sustainable production of food and feed.

**Abstract:**

Insects are a potential substitute for conventional meat and can be part of a sustainable human diet due to their valuable nutrients and relatively low environmental production impact. One species that is already produced for human consumption and livestock feed is the mealworm, i.e., larvae of *Tenebrio molitor*. Knowledge of the effects of temperature, and particularly photoperiod, on mealworm development is scarce, but crucial for the improvement of rearing. Therefore, the effects of three temperatures (20 °C, 25 °C, and 30 °C), in combination with three photoperiods (long-day—16 h:8 h light:dark; short-day—8 h:16 h light:dark, and constant darkness) on mealworm survival, developmental time, and growth rate were tested. We describe a significant effect of temperature on survival rate, developmental time, and growth rate. Furthermore, significant effects of photoperiod on developmental time and growth rate were found. At 25 and 30 °C and constant darkness, the highest survival and growth rate, along with the shortest developmental time, were observed. Our data can be used to improve the mass rearing of mealworms for an efficient production of food and feed.

## 1. Introduction

Insect farming for human consumption is a growing economic sector [1]. The mealworm, i.e., larvae of *Tenebrio molitor* L. (Coleoptera, Tenebrionidae), is widely used for food and feed production [2,3]. Rearing of this species has several advantages as compared to conventional livestock rearing, e.g., an efficient transformation of feed into edible biomass [4,5], or a lower environmental impact, e.g., CO_2_ production [6,7]. Moreover, mealworms contain high amounts of proteins, essential amino acids, favorable fatty acids, minerals, and vitamins, and also have a high energy content [8].

*Tenebrio molitor* has a worldwide distribution. Under natural conditions, it inhabits, for example, rotten wood and other dead organic matter, or sheltered environments like bird nests or burrows. Furthermore, it is a synanthropic, omnivorous species, feeding on stored products. Generally, it prefers dark environments and is negative phototactic [9,10,11].

Knowledge of abiotic parameters on the development of *T. molitor* is essential for leveraging mealworms for future meat consumption. For example, photoperiod influences mealworm development and growth [12], and the authors in [13] describe significantly shorter larval developmental times and longer pupal periods under long-day conditions (14 h light, 10 h dark) as compared to other photoperiods. Photoperiod also influences eclosion rates with the lowest values at 10 h light, 14 h dark [13].

In addition, [14] found shorter larval developmental times at 25 °C with approximately 150 days, compared to 30 °C with 160–213 days; however, details on experimental conditions are often scarce. The optimum temperature for *T. molitor* is described between 22 and 28 °C [15]. The authors in [15] tested *T. molitor* egg, larval, pupal and adult survival at different temperatures, with 25 °C as the optimal temperature for high survival rates. Temperatures of 10 °C and 35 °C, however, were described as unfavorable, since survival rates decreased at these temperatures [15]. Furthermore, larval survival was found to increase with larval age [16]. The authors in [13,17] found significantly different mealworm developmental times at different temperatures, with the fastest larval development of about 111 days at 30 °C and about 127 days at 27.5 °C, respectively. At 31 °C, [18] recorded the highest mealworm wet mass growth per day and the highest metabolic rate; the highest energy conversion efficiency occurred at 23.3 °C. 

Although *T. molitor* is widely used for food and feed production [2], certain aspects of optimum rearing conditions are largely unknown. In particular, specific knowledge of photoperiod on development is lacking. This study aims to shed light on the effects of temperature and photoperiod on mealworm development. Three different temperatures (20 °C, 25 °C, and 30 °C) in combination with three photoperiods (long-day LD 16 h light:8 h dark, short day SD 8 h:16 h, and constant darkness 24D 0 h:24 h) were tested to assess the effects of abiotic factors on survival rate, developmental time, and growth rate of mealworms. This data will help to increase the farming efficiency of *T. molitor* and can contribute to a sustainable meat production.

## 2. Materials and Methods 

### 2.1. Experimental Setup

*Tenebrio molitor* used in this study originated from a mass rearing from LarveMe (formerly dieWurmfarm, Bad Sankt Leonhard, Carinthia, Austria). Subsequently, a stock culture with freshly hatched adult beetles was maintained at 22 °C and daylight in a plastic box with aeration slits and filled with about 2 cm of feed (lucerne 8%, maize meal 12%, beer yeast 10%, wheat bran 70%).

Adult beetles used for our experiments were sexed based on differences in the abdominal sternites [19]. First, one female and one male *T. molitor* adult, randomly selected from the stock culture, were put in a plastic box (9 × 6 × 5 cm) (with a lid containing aeration holes) to initiate mating and oviposition. One cm of feed was filled into the box, and ten boxes each were put in different incubators under experimental conditions: three temperature regimes, i.e., 20 °C, 25 °C, and 30 °C, and three different photoperiods, i.e., long day LD (16 h light L, 8 h dark D), short day SD (8 h L, 16 h D), and constant darkness 24D (0 h L, 24 h D). After three weeks, i.e., when oviposition was completed, adults were removed from the plastic boxes. Subsequently, the presence of young larvae was monitored by screening the boxes for instars four to six (according to head capsule width, as described by [20]; L1 to L3 instars were hardly visible in the feed) on a daily basis. 

Afterwards, 20 fourth to sixth instar larvae were randomly selected for experimental trials. Then, we measured their total weight and transferred them to new boxes filled with 1 cm of fresh feed before exposing them to the conditions described above (i.e., ten plastic boxes with twenty mealworms each per condition). 

Dead larvae and pupae were removed from experimental boxes weekly to record survival rates. Furthermore, larval weight was assessed once per week to calculate growth rates and head capsule width, in order to monitor instar development, until pupation occurred. Survival rates and growth rates were recorded from fourth to sixth instar larvae until 95% of the larvae per box pupated. Developmental time was recorded from the larval eclosion from the eggs until pupation.

### 2.2. Data Analysis

To compare mean survival rates, mean developmental times, and mean growth rates among experimental conditions, a two-way ANOVA was performed, with Tukey tests as post-hoc tests (α = 0.05). The normal distribution of data was assessed by conducting a Shapiro–Wilk normality test. All analyses were conducted with SPSS Statistics v. 27. Details on the calculations of survival rate, developmental time, and growth rate are provided in File S1.

## 3. Results

### 3.1. Survival Rate

**Figure 1:**
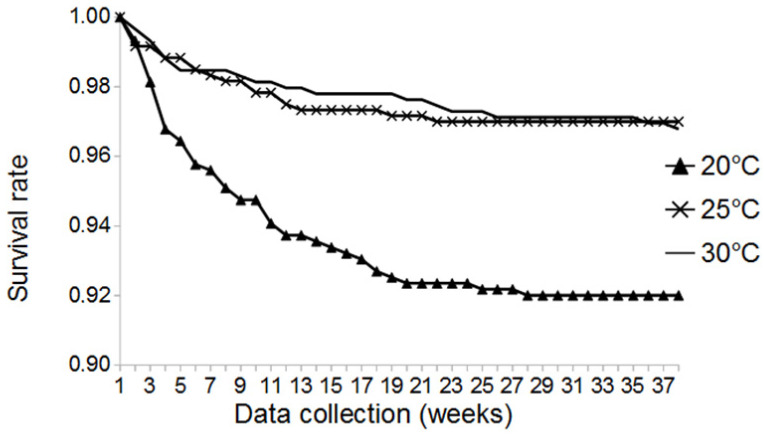
Survival rates (in %) of mealworms at three different temperature regimes (20 °C, 25 °C, and 30 °C).

We found a significant effect of temperature on the survival of mealworms (*p* = 0.001). In general, survival rates among all treatments were high, with mortality rates of less than 10% over a period of 37 weeks (Figure 1). Across all photoperiods tested, there was a significant difference between the survival rate at 20 °C, with a mean value of 92.0%, and the mean survival rate at 25 °C, with 97.0% (*p* = 0.003), as well as between mean values at 20 °C and those at 30 °C, with 96.7% (*p* = 0.006) (Table S1). There was no significant difference between mean survival rates at 25 °C and 30 °C (*p* = 0.978). The lowest survival rate was recorded at 20 °C/24D, with a mean survival rate of 90.4%. The highest survival rate was recorded at 25 °C/24D and 30 °C/24D, both with a mean survival rate of 98.5%. 

When comparing survival rates at LD, SD, and 24D at the various temperature conditions, there was no significant difference of mean values among photoperiodic conditions (Table S4). Furthermore, no significant interaction between temperature and photoperiod on survival rate was found (*p* = 0.237). The influence of temperature on survival was further corroborated, as we found significant differences among temperature conditions within the various photoperiodic treatments (Tables S1 and S4).

### 3.2. Developmental Time

**Figure 2:**
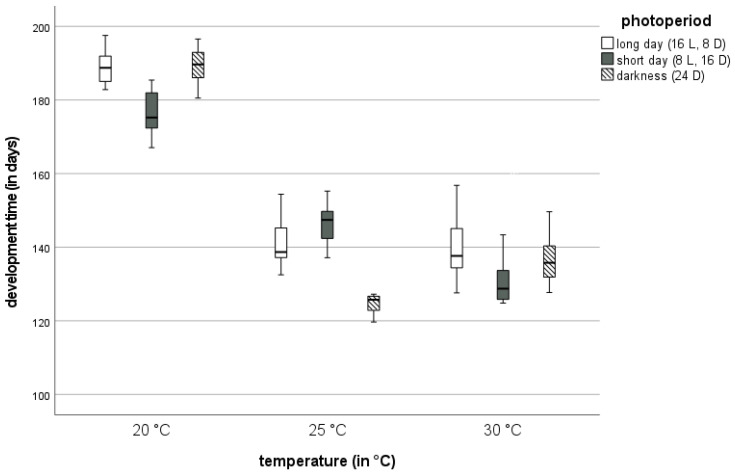
Developmental times (in days) of mealworms at three different temperatures (20 °C, 25 °C, and 30 °C), grouped by three photoperiods: long day (LD), short day (SD), and constant darkness (24D).

**Figure 3:**
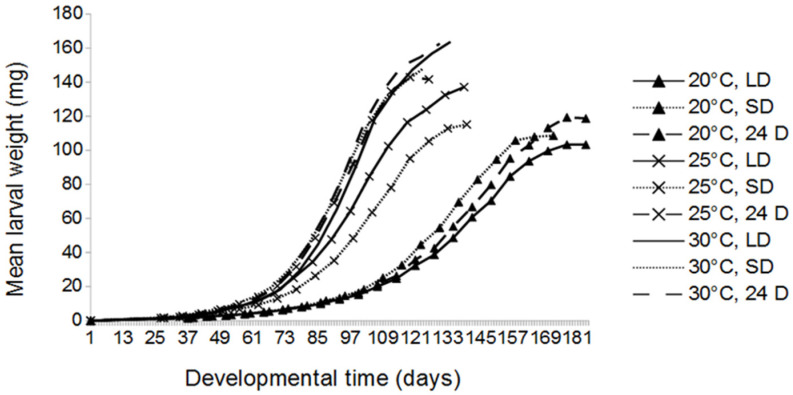
Mean larval weight (in mg) during development (in days) of mealworms at 20 °C, 25 °C, and 30 °C temperatures and LD, SD, and 24D photoperiods. The slopes of curves represent growth rates of all mealworms in one incubator.

We found a significant influence of temperature on larval developmental time, i.e., from the larval eclosion from eggs until pupation (*p* < 0.001). Across all photoperiods tested, the developmental time of mealworms at 20 °C, with a mean of 184.8 days, was significantly higher than at 25 °C and 30 °C, respectively (both *p* < 0.001) (Figure 2 and Figure 3, Table S2). There was no significant difference of mean developmental times between 25 °C, with a mean value of 138 days, and 30 °C, with a mean of 136.1 days (*p* = 0.558). 

There was a significant effect of photoperiod on the developmental time of mealworms (*p* = 0.001), as well as a significant interaction between temperature and photoperiod (*p* < 0.001). Across all temperatures, the mean developmental time under LD with 156.7 days was significantly higher than under SD (*p* = 0.016) and 24D (*p* = 0.001), respectively (Figure 2, Table S2). There was no significant difference between mean values at SD with 151.9 days and 24D with 150.3 days (*p* = 0.632). In general, mealworm developmental time was shorter under SD or 24D (Table S2). 

At 20 °C, the lowest mean values for developmental time were observed under SD conditions. The longest developmental times occurred at 20 °C/LD and 20 °C/24D, both with about 189 days (Table S2). At 25 °C, the lowest mean developmental times were found at 24D. At 30 °C, there were no significant differences in developmental time between different photoperiods (Table S5). At LD, mealworms developed slower at 20 °C, compared to 25 °C and 30 °C. At SD, there was a decline in developmental times with rising temperatures. At 24D, the lowest developmental time with 125.6 days was recorded at 25 °C, which was the shortest developmental time over all the experimental trials (Table S2). 

### 3.3. Growth Rate

**Figure 4:**
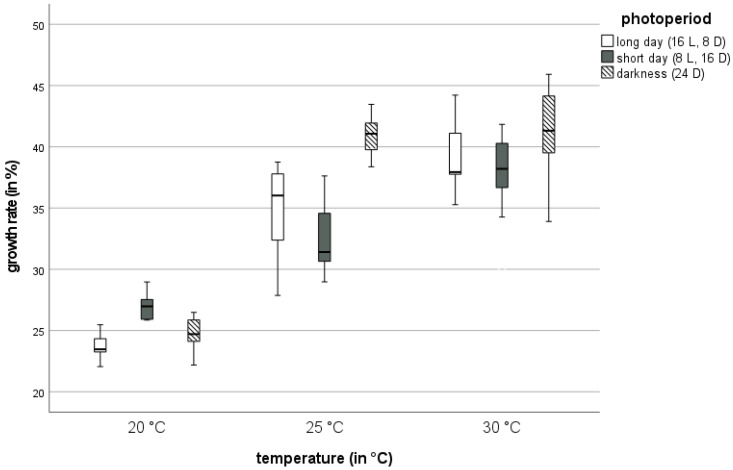
Mean growth rates (in %) of mealworms at three different temperatures (20 °C, 25 °C, and 30 °C), grouped by three photoperiods: long day (LD), short day (SD), and constant darkness (24D).

Temperature had a significant effect on the growth of *T. molitor* larvae (*p* < 0.001). Across all photoperiods, growth rates among the three temperature regimes were significantly different from each other (*p* < 0.001 each). The mean growth rate at 20 °C was the lowest with 25.1%, followed by 25 °C with 36.2%, and 30 °C being the highest with 39.2% (Figure 4, Tables S3 and S6).

There was also a significant effect of photoperiod on the growth rate (*p* < 0.001), as well as a significant interaction between temperature and photoperiod (*p* < 0.001). Across all temperatures, the growth rate under 24D (mean 35.7%) was significantly higher than the growth rate under LD (32.5%) or SD (32.3%), (both *p* < 0.001). However, there was no significant difference between growth rates under LD and SD (*p* = 0.962). Mealworms at 25 °C or 30 °C gained more weight in the same time period under 24D as compared to LD or SD. The highest growth rates were recorded at 25 °C and 30 °C and 24D, with means of 41.2% and 41.1%, respectively. The lowest growth rate, i.e., 23.7%, was observed at 20 °C and LD (Table S6). 

At 20 °C, the highest mealworm growth rate occurred under SD. At 25 °C and 30 °C, the highest growth rate occurred under 24D. At LD and SD, there was an increase in growth rates with rising temperature. At 24D, there was a significant difference between 20 °C and the two other temperature regimes, but the mealworm growth rate at 25 °C and 30 °C was similar (Table S6).

## 4. Discussion

### 4.1. Survival Rate

There was a significant influence of temperature on the survival rates of mealworms. Mealworms died more frequently at 20 °C, with a mean survival rate of about 92%, compared to the two other temperature regimes, with a survival rate of about 97%. Generally, more mealworms died at the beginning of the experiment, and older larvae had a higher survival rate. The authors of [15] described that young mealworms are more susceptible to temperature extremes than older larvae; however, with no difference in mortality among larval instars at 25 °C. The authors of [4] recorded a survival rate of 86%, from hatch day until pupation, at 28 °C. This survival rate is about 10% lower than the survival rates reported here. This might be related to the experimental setup, as [4] used first instar larvae; therefore, a higher mortality of young instars was likely observed. These differences between larval age (especially of young larvae) could not be observed in this study, as data collection began with instar four to six, with a larval age of about four to five weeks. Several studies reported high survival rates, above 90%, at temperatures of 25–30 °C [15,21,22]; others recorded lower values of 70–84% under similar conditions [23,24,25]. In comparison, differences in the survival rates at 25 °C and 30 °C in our study might be related to different starting points of data collection and to different feeds.

Results from the experiments presented here confirm that higher survival rates are observed at higher temperatures, with an optimal rearing temperature between 25 °C and 30 °C. Moreover, [15,17] state that the temperature preference of *T. molitor* is between 22–28 °C, and that temperatures above 35 °C and below 20 °C are associated with decreasing survival rates and increasing stress.

### 4.2. Developmental Time

Here, a significant influence of temperature and photoperiod on the developmental time of mealworms was found. In general, we describe shorter developmental times at higher temperatures, with no significant difference between 25 °C and 30 °C. The lowest developmental time of about 125 days was recorded at 25 °C and 24D. Rearing under LD resulted in significantly longer developmental times than under SD and 24D. At all three temperature regimes, the developmental time was similar to those observed in previous studies [17]. Moreover, the difference of developmental time between 20 °C and 25 °C was significantly greater than the difference between 25 °C and 30 °C, which is in accordance with other work [17]. The authors of [26] found the lowest developmental times at 25 °C and considerably higher developmental times at 30 °C. In general, there is a great range of developmental time values of mealworms across different studies. For example, low values at 25 °C and 30 °C were reported from studies with favorable, protein-rich feed and a water source [4,23,24,27]. Upper values originate from studies with unfavorable feed and no water source [23,24]. Further, differences can originate from the definition of ‘developmental time’ across different studies. 

Our study showed a significantly higher developmental time under LD conditions compared to the other photoperiods. At 25 °C and 30 °C, developmental times under 24D were significantly lower, compared to LD or SD. However, the differences in developmental time between different photoperiods were small, i.e., the influence of photoperiod was low compared to temperature. The authors of [9,16] state that mealworms avoid daylight and are more active at night. However, no data on mealworm activity at constant darkness (24D) are currently available. Our analyses show that a faster development is observed at shorter day-length.

### 4.3. Growth Rate

We report a significant influence of temperature and photoperiod on the growth rates of mealworms. Results show higher growth rates at higher temperatures. Rearing under 24D resulted in a significantly higher growth rate than under LD and SD conditions. Weekly growth rates of mealworms were similar to [4,27], who recorded larval weights at 25 °C and 28 °C, respectively; these weekly growth rates were higher than the values reported here. The authors of [22] found a daily growth rate of 4–7% at 27 °C over the entire larval stage, which is in accordance with a daily growth rate of 5% at 25 °C reported in this study. In [18] daily growth rates of mealworms at 19 °C, 25 °C, and 31 °C between 25–75% were recorded. The authors found significantly higher growth rates at higher temperatures, with a peak of 17% daily growth rate at 31 °C, also being in accordance with our study. The influence of photoperiod on the growth rate of mealworms is relatively low, with a significantly higher growth rate at constant darkness than under light conditions. In a recent study, no significant differences of growth rates between SD and 24D conditions were reported [11]. However, there are likely other additional factors that affect mealworm growth, such as feed, density of individuals, or humidity.

Taken together, our study provides important data on the development, growth, and survival of *T. molitor* larvae at different temperature and photoperiodic conditions. In general, at higher temperature regimes and constant darkness, higher survival and growth rates, as well as shorter developmental times, were observed. These data can be used to increase farming efficiency and contribute to a sustainable production of mealworms for food and feed.

## Data Availability

All data are provided in the Supplementary Materials.

## Supplementary Materials

The following are available online at https://www.mdpi.com/article/10.3390/insects13040321/s1, File S1: Calculations of survival rate, developmental time, and growth rate. File S2: Results of survival rates (Table S1), developmental times (Table S2), and growth rates (Table S3) of *Tenebrio molitor* larvae at 20 °C, 25 °C, and 30 °C, and long-day LD, short-day SD, and constant darkness 24D. Table S4: Significance values among mean survival rates at 20 °C, 25 °C, and 30 °C, and long-day LD, short-day SD, and constant darkness 24D. Table S5: Significance values among mean developmental times at 20 °C, 25 °C, and 30 °C, and long-day LD, short-day SD, and constant darkness 24D. Table S6: Significance values among mean growth rates at 20 °C, 25 °C, and 30 °C, and long-day LD, short-day SD, and constant darkness 24D.
