# Supplementary material for: Effect of Temperature and Photoperiod on Development, Survival, and Growth Rate of Mealworms, Tenebrio molitor
Source: Insects. 2022 Mar 24;13(4):321. doi: 10.3390/insects13040321 (PMC9029539; doi:10.3390/insects13040321)
Supplement: Supplementary file 1 [file insects-13-00321-s001.zip › File S1.pdf]

File S1

Details on calculations of survival rate, developmental time, and growth rate of *Tenebrio molitor* larvae.

Survival rate: The number of all pupated larvae, divided by the number of L4-L6 larvae, at the beginning of the experiment per plastic box (n).

Developmental time: The developmental time of each mealworm, from the larval eclosion from eggs to pupation, was recorded in days. To calculate the average developmental time per plastic box (n), the mean of all larvae per box was calculated.

Growth rate: The growth rate of L4 to L6 mealworms was calculated until 95% of the larvae per box pupated. Every week, the weight of all living larvae per box was measured, and this value was divided by the number of living larvae to calculate the average larval weight. The average larval weight per box of week k_1 was subtracted by the average larval weight per box of week k_0 to obtain the wet mass increase (of the average larva) in week k_1.

$$k_0 = \text{week } 0, k_1 = \text{week } 1, k_x = \text{last week of data collection}, gr = \text{growth rate}$$

$$\text{wet mass increase } k_1 = \text{average larva weight } k_1 - \text{average larva weight } k_0 \text{ (in mg)} \quad (1)$$

The wet mass increase in week k_1 was divided by the average larval weight of week k_0 to obtain the growth rate of week k_1. This was multiplied by 100 to obtain the growth rate of the average larva per box per week in %.

$$gr\ k_1 = \frac{\text{wet mass increase } k_1}{\text{average larva weight } k_0} * 100 \text{ (in \%)} \quad (2)$$

The calculation of the mean growth rate per box was weighted by the number of living larvae per week.

$$n = \frac{gr\ k_1 * \text{number of living larvae } k_1 + gr\ k_2 * \text{number of living larvae } k_2 + \ldots + gr\ k_x * \text{number of living larvae } k_x}{\text{number of living larvae } k_1 + \text{number of living larvae } k_2 + \ldots + \text{number of living larvae } k_x} \text{ (in \%)} \quad (3)$$

This was calculated until 95% pupation per box to reduce the influence of a possible negative growth rate during the pupation time.
