# Supplementary material for: Effect of Temperature and Photoperiod on Development, Survival, and Growth Rate of Mealworms, Tenebrio molitor
Source: Insects. 2022 Mar 24;13(4):321. doi: 10.3390/insects13040321 (PMC9029539; doi:10.3390/insects13040321)
Supplement: Supplementary file 1 [file insects-13-00321-s001.zip › File S2.pdf]

File S2

Results of survival rates, developmental times, and growth rates of *Tenebrio molitor* larvae at 20 °C, 25 °C, and 30 °C, and long-day LD, short-day SD, and constant darkness 24D.

Table S1 Survival rates (mean values ± standard deviation in %) of mealworms at 20 °C, 25 °C, and 30 °C and LD, SD, and 24D photoperiods. n = number of plastic boxes per treatment; individuals = total number of investigated larvae. For detail on significant differences, see Supplement 3.

Temperature	Photoperiod	Mean ± SD	n	Individuals
20 °C	LD	93.8 ± 5.8	10	192
	SD	91.9 ± 7.6	10	199
	24D	90.4 ± 7.6	10	196
25 °C	LD	96.5 ± 4.7	10	200
	SD	96 ± 5.2	10	200
	24D	98.5 ± 2.4	10	199
30 °C	LD	94.1 ± 7.7	10	196
	SD	97.5 ± 3.6	10	198
	24D	98.5 ± 3.4	10	199
20 °C	All	**92 ± 7.0**	30	587
25 °C	All	**97 ± 4.3**	30	599
30 °C	Alll	**96.7 ± 5.4**	30	593
All	LD	**94.8 ± 6.1**	30	588
	SD	**95.1 ± 6**	30	597
	24D	**95.8 ± 6.2**	30	594
	Overall mean	**95.2 ± 6**	90	1779

Table S2 Developmental time (mean values ± standard deviation in days) of mealworms at three temperatures (20 °C, 25 °C, and 30 °C) and three photoperiods (long day LD, short day SD, and darkness 24D). n = number of plastic boxes per treatment; individuals = total number of investigated larvae. For details on significant differences among experimental conditions, see Supplement 4.

Temperature	Photoperiod	Mean ± SD	n	Individuals
20 °C	LD	188.9 ± 4.5	10	180
	SD	176.4 ± 5.9	10	183
	24D	189 ± 5.2	10	177
25 °C	LD	141.5 ± 7.5	10	193
	SD	146.8 ± 5.6	10	192
	24D	125.6 ± 4.2	10	196
30 °C	LD	139.8 ± 8.3	10	185
	SD	132.5 ± 10.1	10	193
	24D	136.3 ± 6.4	10	196
20 °C	Alll	**184.8 ± 7.9**	30	540
25 °C	All	**138 ± 10.8**	30	581
30 °C	All	**136.2 ± 8.7**	30	574
All	LD	**156.7 ± 24.1**	30	558
	SD	**151.9 ± 20**	30	568

	24D	**150.3 ± 28.7**	30	569
	Overall mean	**153 ± 24.4**	90	1695

Table S3 Growth rate (mean values ± standard deviation in %) of mealworms at three temperature regimes (20 °C, 25 °C, and 30 °C) and three photoperiods long day LD, short day SD, and darkness 24D. n = number of plastic boxes per treatment; individuals = total number of investigated larvae. For details on significant differences among experimental conditions, see Supplement 5.

**Temperature**	**Photoperiod**	**Mean ± SD**	**n**	**Individuals**
20 °C	LD	23.7 ± 1.1	10	171
	SD	27 ± 1.1	10	177
	24D	24.8 ± 1.3	10	169
25 °C	LD	35.1 ± 3.4	10	186
	SD	32.3 ± 2.6	10	183
	24D	41.2 ± 1.6	10	188
30 °C	LD	38.8 ± 2.7	10	177
	SD	37.7 ± 3.5	10	184
	24D	41.1 ± 3.7	10	188
20 °C	All	**25.1 ± 1.8**	30	517
25 °C	All	**36.2 ± 4.5**	30	557
30 °C	All	**39.2 ± 3.5**	30	549
All	LD	**32.5 ± 7**	30	534
	SD	**32.3 ± 5.1**	30	544
	24D	**35.7 ± 8.2**	30	545
	Overall mean	**33.5 ± 7.0**	90	1623
